# Dr. C. R. Sutton

**Published:** 1913-11

**Authors:** 


Dr. C. R. Sutton (not listed in State Directory), an eye specialist of Warsaw, died October 4, aged 53. He came to Warsaw from Perry in 1910.

